# Titanium dioxide nanoparticles alleviate phosphorus deficiency stress in apple plants

**DOI:** 10.1038/s41598-025-07987-3

**Published:** 2025-07-08

**Authors:** Sabry Soliman, Yi Wang, Mina S. F. Samaan

**Affiliations:** 1 Department of Horticulture, Faculty of Agriculture, Ain Shams University, Cairo, Egypt; 2 College of Horticulture, China Agriculture University, Beijing, 100193 China

**Keywords:** Titanium dioxide nanoparticles (TiO₂NPs) – Phosphorus (P) deficiency, Apple plants- vegetative and root architecture, CK (CK)- chlorophyll, Plant physiology, Plant signalling, Plant stress responses

## Abstract

Titanium dioxide nanoparticles (TiO₂NPs) can influence various physiological processes in plant tissues. Phosphorus (P) deficiency can cause severely impair growth which may lead to lethal damage to plants, such as apple trees. The aim of this study was to investigate the role of titanium in nanometric form, in alleviating the consequences of P deficiency. One-year-old MM106 apple rootstock plants were divided into two groups: one was subjected to phosphorus deficiency (-P), while the other received balanced fertilization (+ P). Both groups were sprayed with TiO₂NPs at concentrations of 0, 10, 50, and 100 mgL⁻¹, along with a normal TiO at 100 mgL⁻¹ The results showed that 10 mgL⁻¹ TiO₂NPs were the most effective at enhancing various physiological and growth parameters in -P plants. These included increased leaf cytokinin (CK) content (isopentyl adenine), chlorophyll levels, root fresh weight, root network area, root length in the 2–6 mm range, and surface area per cm². These improvements contributed to the increased uptake of phosphorus, iron, and copper, which are essential for chlorophyll synthesis. The interplay of these factors, along with increased CK levels, improved vegetative growth and mitigated the adverse effects of phosphorus deficiency.

**Supplementary Information:**

The online version contains supplementary material available at 10.1038/s41598-025-07987-3.

## Introduction

Phosphorus (P) is a vital macronutrient essential for plant growth and development, playing a pivotal role in energy transfer, signal transduction, and the biosynthesis of nucleic acids, phospholipids, and ATP^1–10^. Its deficiency, common in many soils due to poor mobility and availability, severely limits photosynthesis, chlorophyll content, cytokinin (CK) regulation, and root development, thereby reducing crop productivity^5,11–27^. In apple (*Malus domestica* Borkh.), a high-value fruit crop, P deficiency is particularly detrimental, affecting root architecture, nutrient uptake, and photosynthetic efficiency.

While phosphate fertilizers are commonly used to address P deficiency, they are inefficient and environmentally unsustainable, often resulting in nutrient runoff and water eutrophication^28–30^. Consequently, there is a growing interest in nanotechnology-based solutions that improve nutrient use efficiency and sustainability. Titanium dioxide nanoparticles (TiO₂NPs), in particular, have emerged as promising tools due to their unique physicochemical properties and bio-interactive capabilities^31–37^.

TiO₂NPs have shown potential to alleviate P deficiency by enhancing phosphate solubility, stimulating phosphatase activity, improving root growth, and influencing soil–plant nutrient dynamics^38,39^. Additionally, they may modulate antioxidant activity, photosynthetic capacity, and expression of P transporter genes, but their effects on hormonal pathways, particularly CKs, under P-deficient conditions in perennial fruit crops remain largely unexplored^2,32,36^.

This study presents an innovative approach by exploring, for the first time, the role of TiO₂NPs in modulating cytokinin levels, chlorophyll synthesis, and root morphology in apple trees under phosphorus stress. By integrating nanotechnology with hormonal and physiological analyses, it reveals novel insights into how TiO₂NPs can enhance P uptake and improve plant resilience. This research offers a sustainable and cutting-edge strategy for mitigating nutrient deficiencies in horticulture, highlighting TiO₂NPs as potential next-generation agricultural amendments.

## Materials and methods

### Plant materials and experimental design and conditions

The aim of this study was to investigate the ability of the foliar application of TiO₂NPs to alleviate the impact of P deficiency. Bare-root apple transplants of one-year-old MM106 apple rootstock (*Malus × domestica Borkh*) were used in the present study. The plant rootstocks transplants (plant materials) used in this study were obtained from the Agricultural Research Center (ARC), which operates under the Ministry of Agriculture and Land Reclamation, Egypt. The experiment was conducted in a saran house belonging to the Horticulture Department, Faculty of Agriculture, Ain Shams University, Cairo, Egypt. Due to recent climate changes, temperatures in Egypt have warm autumns and winters and early spring initiation. The study was carried out from the last week of February, while the end of the experiment was in the last week of December; however, sampling occurred neither at the beginning nor at the end of the experiment but during peak leaf development to avoid senescent tissues. Leaf sampling was carried out during the active growth phase, targeting fresh, physiologically active leaves. The experiment was conducted in a greenhouse, and it began last week using bare-root apple transplants. The greenhouse ambient temperatures during the study during all the experimental period ranged from 15 to 35°C, with both day and night temperatures being considered. The relative humidity ranged from 50–80%, and the transplants were exposed to 70% light intensity and normal photoperiods. To exclude salt and avoid confounding effects from organic matter, plants were cultivated in washed sandy soil without organic amendments.The transplants were treated with different concentrations of TiO₂NPs with or without phosphorous deficiency stress.

The experiment was a factorial design, and the treatments were arranged in a split-plot design (SPD). The main factor (phosphorous ±) was placed in the main plots, and the second factor (TiO₂ and TiO₂NP levels) was placed in the submain plots. The main plots were divided into two groups: the first group received the complete nutrition including Phosphorus (+ P) according to Hoagland solution^40,41^, while the second group received the same nutrition solution without phosphorus (-P). The nutrition solutions (fertilization) were applied with the irrigation three times per week, and it treated six weeks before the TiO₂NPs application. Each main plot was divided into five submain plots for foliar application by TiO₂ and TiO₂NPs. The plants sprayed by TiO₂ and TiO₂NPs three times, one per week for three weeks. The normal (mineral) TiO₂ was used as a second control to identify the nanoparticle effects. The treatments of Titanium 0were 0, 100 mgL⁻¹ of TiO₂ and10, 50 and 100 mgL⁻¹ of TiO₂NPs^36^. The concentration of TiO₂NPs were use1d as categorical levels^36^. Each treatment included five replicates, and each replicate represented one transplant. The treatments were as follows:

- _+_P + distilled water (+ P control, or 0 mg L⁻¹).
- _+_P + TiO₂ (titanium dioxide) 100 mgL⁻¹.
- _+_P + TiO₂NPs 10 mgL^-1^.
- _+_P + TiO₂NPs 50 mgL⁻¹.
- _+_P + TiO₂NPs 100 mgL⁻¹.
-P + distilled water (-P control, or 0 mg L⁻¹).
-P + TiO₂ (titanium dioxide) 100 mgL⁻¹.
-P + TiO₂NPs 10 mgL⁻¹.
-P + TiO₂NPs 50 mgL⁻¹.
-P + TiO₂NPs 100 mgL⁻¹.

While_+_P = with P fertilization, -P = without P fertilization (P deficiency), TiO₂NPs = TiO₂ nanoparticles, TiO₂ (titanium dioxide) = mineral titanium dioxide at 100 mgL⁻¹.

### TiO₂NPs preparation, concentrations, and applications

The TiO₂ nanoparticles were purchased from US Research Nanomaterials, Inc., and the product number was US1019F, CAS#: 13463-67-7^42^. TiO₂NPs were used in this study with a purity of 99+%. The nanoparticles had an average particle size (APS), and median particle size (D50) of 20 nm, a specific surface area (SSA) ranging from 10 to 45 m²/g, and a bulk density of 0.46 g/mL. The pH of the nanoparticle suspension was measured between 5.5 and 6.0. The material appeared as a white powder. The loss of weight in drying was 0.48%, while the loss of weight on ignition was 0.99%.

The powder was weighed at each concentration, and then carboxymethylcellulose (CMC) was added to the solution at a rate of 1.5 gl^− 1^ to avoid the formation of titanium nanoparticle precipitates. The TiO₂ NP suspension was applied as a foliar spray using a handheld sprayer in the greenhouse, with a spray volume of 1–3 L per 10 m². Droplets were targeted to fall within the size range of 150–250 μm to ensure uniform coverage of both the upper and lower leaf surfaces while minimizing drift. The Hand sprayers with different concentrations were used to spray the transplants until they covered the entire foliage. Measurements were carried out twice; before and after of treatment of TiO₂NPs.

### Vegetative parameters

All replicates of the apple trees were measured for plant height and twig length with a measuring tape. The number of shoots and leaves per replicate were counted. Stem thickness and leaf dimensions (length and width) were measured with a digital caliper. Mature leaves midway up the shoot were used to measure the leaf length and width. The leaf area was calculated according to Boyacı and Küçükönder^43^. The fresh weight, dry weight and biomass of the roots were measured. The root samples were collected, cleaned, washed, and then separated into two groups. The first group was prepared by splitting the roots for analysis. The other groups of samples were dried in a drying oven at 70 °C until the weight of each sample reached zero.

### Chemical parameters

#### SPAD index (chlorophyll index)

The SPAD reading index of the mature leaves was recorded via a chlorophyll meter (Konica Minolta SPAD 502 Plus Chlorophyll Meter, INC, Osaka, Japan). Five random leaves were chosen per replicate to determine the chlorophyll SPAD index.

#### Leaf mineral contents

Several mature leaves at the midway part of the stem were collected and dried at 70 °C until the weight settled. Then, 1 g of dried leaves were weighed, wet-digested in 20 ml of sulfuric acid tell the complete digestion of the dried leaves, and filled with 50 ml of H_2_O_2_. A Kjeldahl distillation instrument (Kjeltec™ 8400, Foss, Hilleroed, Denmark) was then used to measure the total nitrogen content in the leaves. The other minerals were determined by a flame photometer, UV-Vis spectrophotometer, and Atomic absorption spectrophotometer (ASS). The titanium content in the leaves was measured via an ICP optical emission spectrometer (ICP-OES) (Ultima Expert, HORBIA).

Measurements were carried out twice; before and after of treatment of TiO₂NPs an.

#### Cytokinin content in leaves (zeatin and isopentyl adenine)^44–48^

##### Sample collection

Fresh leaf samples were collected in the early morning and immediately frozen in liquid nitrogen to prevent CK degradation. The frozen samples were ground to a fine powder using a mortar and pestle with liquid nitrogen.

##### Cytokinin extraction

Approximately 1 g of powdered leaf tissue was weighed and placed into a tube. CK were extracted using 10 mL of 80% methanol containing 1% formic acid. The samples were vortexed for 1–2 min and sonicated for 15–20 min in an ultrasonic bath. The extracts were then centrifuged at 10,000 × g for 15 min at 4 °C, and the supernatant was collected and filtered.

##### HPLC analysis

The filtered extracts were analyzed using a Waters 1525 Binary HPLC Pump equipped with a 1.39 × 300 mm C18 reverse-phase column. The mobile phase consisted of:

- Mobile phase A: 0.1% formic acid in water.
- Mobile phase B: Acetonitrile.

A gradient elution was applied, starting with 90% A and 10% B, gradually increasing B to 50% over 30 min. The flow rate was set to 0.8–1.0 mL/min, and CK detection was performed at 254–269 nm using a Waters 2489 UV detector. Waters 2707 autosampler was used to ensure accurate and reproducible sample injections, improving efficiency, minimizing contamination risk, and enabling high-throughput analysis. It provided 20 µM injection volumes, reducing variability between samples and ensuring precise quantification.

### Quantification

CKs were identified by comparing retention times with standard solutions. CK concentrations were quantified using an external standard calibration curve, and the results were expressed as ng/g (mg /100 g) fresh weight.

### Root analysis

Roots were carefully washed with water to remove soil particles and were spread on a flat, contrasting background to minimize overlapping. Root images were analyzed using with ImageJ^49^ and RhizoVision Explorer Version 2.0.3 ^50^. The images were preprocessed by adjusting brightness and contrast, followed by thresholding to enhance root visibility. The following parameters were measured: total root length, root surface area, root volume, average diameter, and the number of root tips and forks. The analyzed data were exported as CSV files for further statistical analysis.

### Statistical analysis

The data were statistically analyzed by One-way analysis of variance (ANOVA) and Duncan’s multiple range test were used to determine the significance of the differences among samples (*p* ≤ 0.05). The factors were considered as the categorial quantitative variables.

## Results

### Morphological Characterization of the TiO₂NPs, and Ti content in leaves

**Fig. 1:**
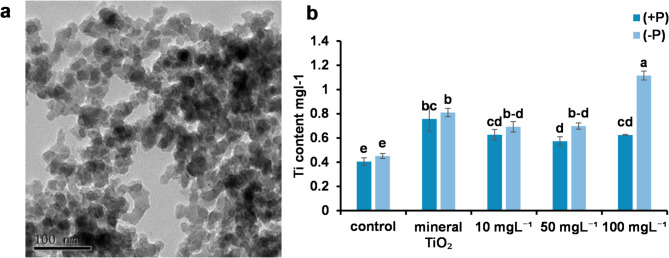
(**a**) Shape and size of the titanium dioxide nanoparticles. This is a transmission electron microscopy (TEM) image of titanium dioxide nanoparticles (TiO₂NPs). The image shows the morphology of the particles, which appear to be aggregated and have irregular, nanoscale shapes. The scale bar at the bottom left indicates that the features are on the order of 100 nanometers (nm). (**b**) Ti content (mg L⁻¹) in the leaf. Different letters indicate significant differences between treatments at the significance level (*P* < 0.05). Error bars represent SEs.

The internal structure, crystallinity, morphology and size distribution at atomic to nanometer scales of TiO₂NPs were analyzed using transmission electron microscopy (TEM) imaging as shown in Fig. 1A. The TEM image revealed that the TiO₂NPs exhibited an irregular morphology, with most particles appearing spherical to slightly angular in shape. The nanoparticles were highly agglomerated, which may be attributed to van der Waals forces or residual synthesis by products.

The observed particle average size of approximately 20 nm, confirming their nanometric nature. The presence of darker regions in the TEM image suggests particle overlapping or increased electron density in certain areas. Overall, the TEM analysis confirmed that the nanoscale dimensions and agglomeration tendency of the synthesized TiO₂NPs, which may influence their surface reactivity and dispersion behavior in biological or environmental applications.

The leaves of -P plants treated with 100 mg L⁻¹ TiO₂NPs presented the highest titanium content, followed by those of plants treated with -P and 100 mg L⁻¹ mineral TiO₂, with a significant difference between the two treatments. This revealed the efficiency of nanoparticles absorption to the leaves. in addition, the increasing in Ti leaf content with -P compared to + P per each treatment indicated that the titanium content in leaves increases significantly under phosphorus deficiency. (Fig. 1)

### The impact of foliar application of TiO₂NPs on the growth and development of apple aerial parts

**Fig. 2:**
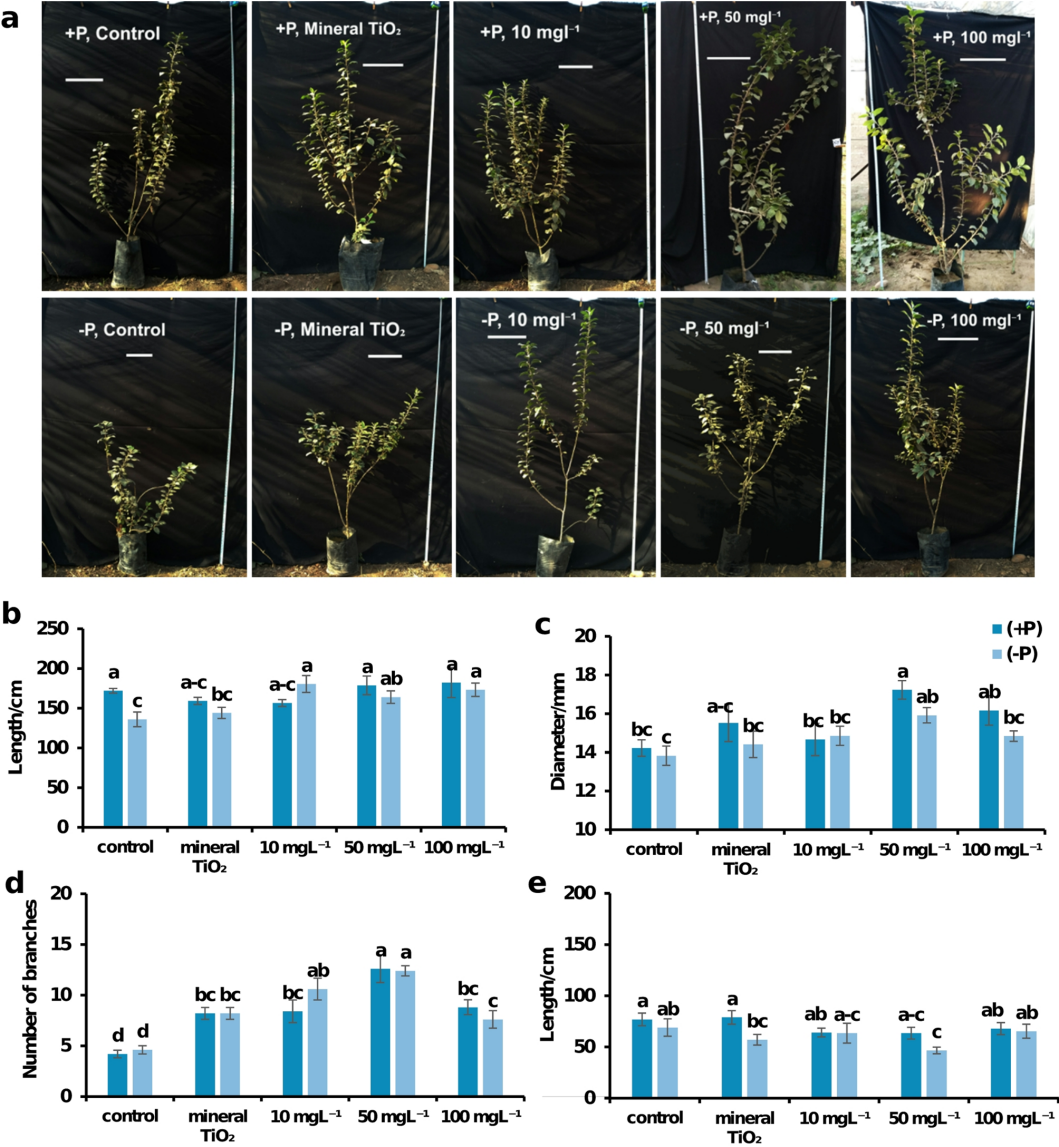
Effects of TiO₂NPs on the height of MM106 apple plants under P deficiency (**a**) Pictures showing only the difference in length among all the treatments. The scale bar indicates that the features are on the order of a 30 cm scale. (**b**) Plant height or shoot length per cm between all the treatments. (**c**) the stem diameter, which reflects the thickness of the main stem per mm. (**d**) Branch count per plant. (**e**) The length of each branch per plant per cm. In all charts, the different alphabet letters indicate significant differences between treatments at the significance level (*P* < 0.05). Error bars represent SEs.

The results indicated that, compared with + P (control), phosphorus deficiency (-P, control) significantly decreased plant height, with the shortest plants found in the -P control group. TiO₂NP treatment had a significant effect on plant height, particularly at a concentration of 100 mgL⁻¹ of TiO₂ nanoparticles (TiO₂NPs). Moreover, no significant difference was observed between the 100 mgL⁻¹ concentration and the other TiO₂NPs concentrations (10 and 50 mgL⁻¹). These findings suggest that TiO₂NPs alleviate the decrease in plant height caused by phosphorus deficiency. (Fig. 2)

The plants treated with 50 mgL⁻¹ of TiO₂NPs presented the greatest stem diameter, closely followed by those treated with 100 mgL⁻¹ TiO₂NPs, with no significant difference between them. Conversely, the control plants presented the thinnest stems, regardless of P fertilization. There was no significant difference in stem diameter between + P and -P plants, irrespective of the TiO₂ treatment. Notably, the combination of + P and 50 mgL⁻¹ TiO₂NPs resulted in the thickest stems, while the thinnest stems were observed in the -P control plants. Interestingly, under phosphorus deficiency, the response did not significantly differ between treatments at 10 mgL⁻¹ and higher concentrations, such as 50 mgL⁻¹ or 100 mgL⁻¹. This highlights a possible threshold effect where increasing concentrations fail to produce additional benefits.“. (Fig. 2)

With respect to the effects of titanium treatment and phosphorus deficiency on branch number and length, +P and -P plants presented similar branch numbers. Interestingly, plants treated with 50 mgL⁻¹ of TiO₂NPs presented the greatest number of branches, regardless of phosphorus fertilization. Notably, +P plants treated with 50 mgL⁻¹ TiO₂NPs presented the highest branch number, followed closely by -P plants subjected to the same treatment, with no significant difference between them. Compared with the control treatment, the treatments with 10, 50, and 100 mgL⁻¹ TiO₂NPs increased the number of branches. However, while the concentration at 10 mg L⁻¹ did not significantly differ from that at 50 mgL⁻¹, it was significantly greater than that at 100 mg L⁻¹⁻¹, suggesting that higher concentrations of TiO₂NPs may negatively impact branching. For branch length, +P control plants and those treated with 100 mg L⁻¹ TiO₂ produced the tallest branches. In contrast, the shortest branches were observed in -P plants treated with 50 mg L⁻¹ TiO₂ NPs, with no significant difference compared with those treated with 10 mg L⁻¹ TiO₂ but were significantly shorter than those treated with 100 mg L⁻¹ TiO₂ NPs. The 50 mg L⁻¹ TiO₂ NPs significantly decreased the branch length, suggesting that they increased branching while reducing the overall branch length (Fig. 2).

One of the key vegetative characteristics in apple growth and development is leaf dimensions and counts. Therefore, the leaf length, width, area, and number of leaves per plant were measured. The results revealed that phosphorus deficiency (-P) decreased leaf characteristics, with a significant reduction in leaf length. However, no significant reductions in leaf width, area, or leaf count were detected when the -P treatments were compared with the + P or control treatments. The lowest significant values for leaf length, width, area, and count were detected in -P plants, both in the control group and in plants treated with 100 mg L⁻¹ TiO₂.

**Fig. 3:**
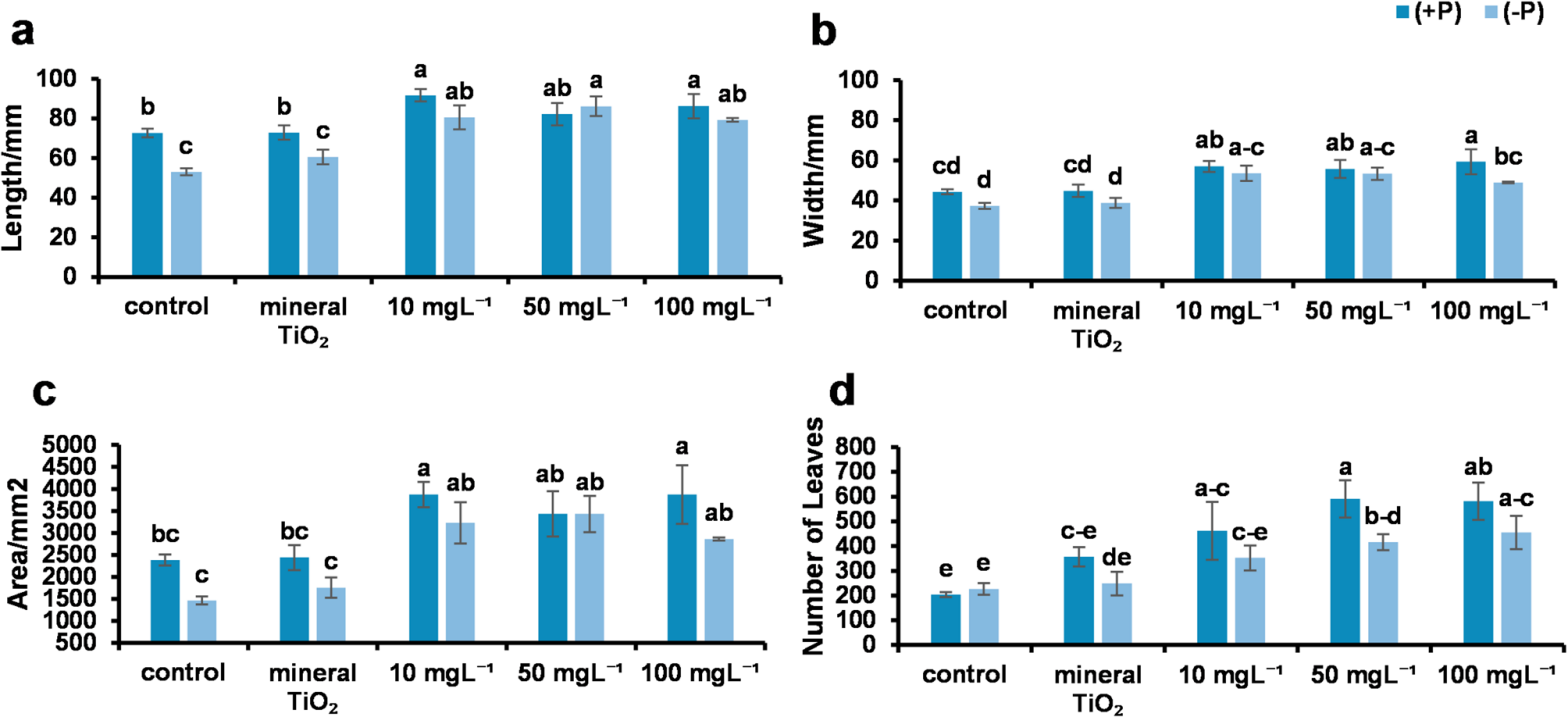
Leaf dimensions: (**a**) Leaf length per mm, (**b**) width and area. (**c**) Leaf area per mm^2^. (**d**) the number of leaves (the number of leaves per plant). In all charts, the different alphabet letters indicate significant differences between treatments at the significance level (*P* < 0.05). Error bars represent SEs.

In contrast, the plants treated with 10 mg L⁻¹ TiO₂NPs, followed by those treated with 50 mg L⁻¹ and 100 mg L⁻¹ TiO₂NPs, presented the greatest differences in leaf length, width, area, and number of leaves regardless of phosphorus fertilization. These findings suggest that TiO₂NPs significantly alleviated the negative effects of phosphorus deficiency on leaf characteristics in the control treatments, enhancing leaf features under both + P and -P conditions. (Fig. 3)

### Effects of tio₂nps on plant root development

Several root architectures and parameters, including the root network area, root length, and root surface area, were measured across different diameter ranges (2–6 mm). -P resulted in a significantly larger root network area than did standard fertilization (+ P), regardless of TiO₂ application. Notably, the greatest root network area was observed in the plants treated with 10 mg L⁻¹ TiO₂NPs, with a significant difference compared with all the other treatments, irrespective of the fertilization regime.

**Fig. 4:**
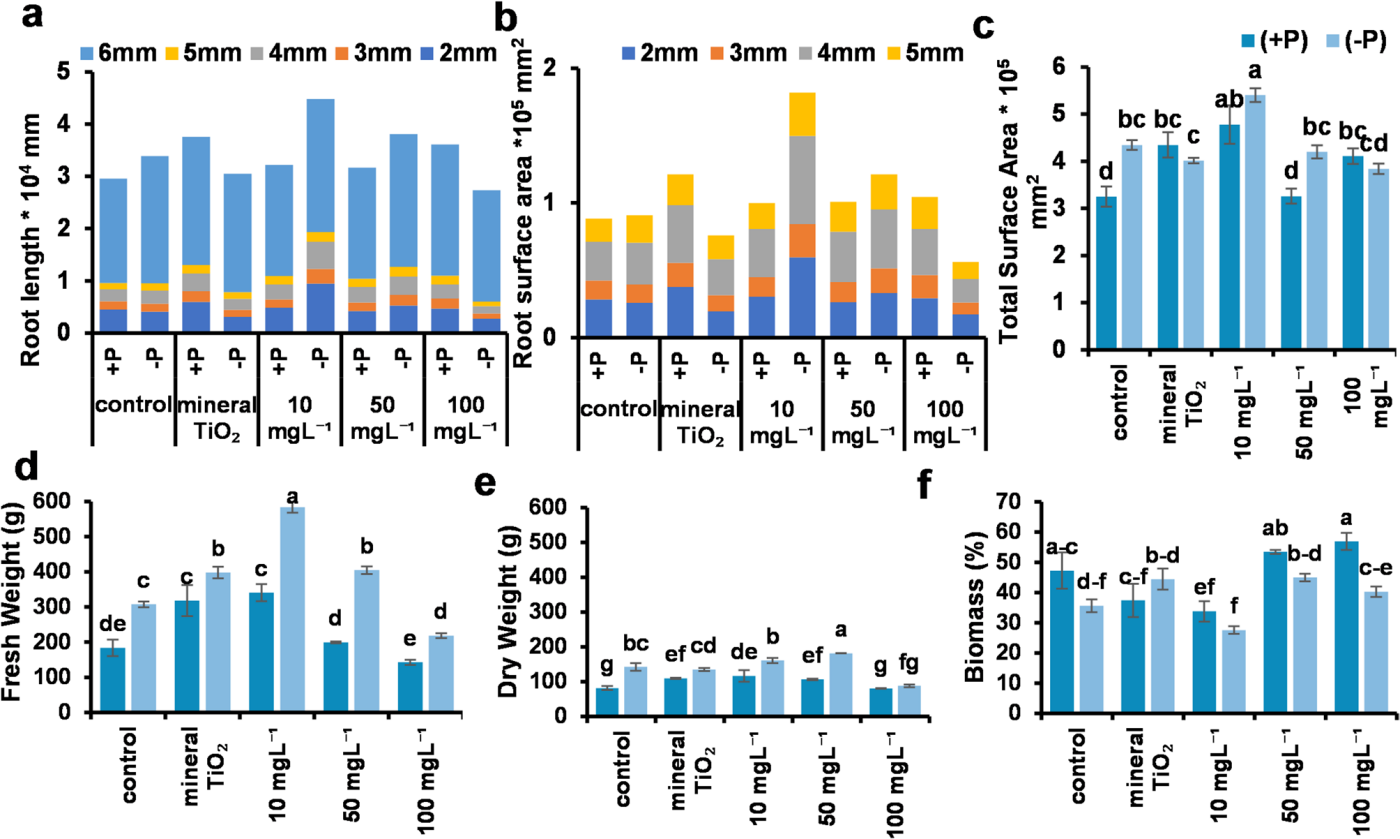
Root characteristics such as root length, surface area, network area, fresh and dry weight, and biomass percentage for roots ranging from 2 to 6 mm in diameter. (**a**) and (**b**) Root surface area per mm^2^. **a**: Differences among all the treatments for all the diameters; the dominant diameter was 6 mm, but (**b**) Focusing of the other diameters from 2 to 5 mm. (**c**) Network area per mm^2^ of the roots between all the treatments. The values of (**a**), (**b**), and (**c**) are multiplied by a power of 10, and the superscript number is the exponent. (**d**) fresh weight per gram (g) (**e**) dry weight per gram (**g**) (**f**) biomass percentage (%). Different letters indicate significant differences between treatments at the significance level (*P* < 0.05). Error bars represent SEs.

The dominant root diameter range for both root length and surface area was 6 mm. No significant differences in root length or surface area within the 2–5 mm diameter range were detected between + P and -P plants, regardless of TiO₂NP application. However, a significant difference was detected in the 6 mm diameter range. The most effective treatment was foliar application of 10 mg L⁻¹ TiO₂NPs, which significantly increased the root length and surface area across all diameter ranges (2–6 mm). (Fig. 4 and supplementary Tables s1-11).

Notably, -P plants treated with 10 mg L⁻¹ TiO₂NPs produced the longest roots across all diameter ranges (2–6 mm) compared with all the other treatments and the control. In terms of root surface area, -P plants treated with 10 mg L⁻¹ TiO₂NPs significantly outperformed those in the 2–5 mm diameter range, except at 5 mm, where no significant difference was detected between -P plants treated with 10 or 50 mg L⁻¹ TiO₂NPs (Fig. 4 and supplementary Tables 1–11).

Fresh and dry root weights were significantly greater in -P plants than in + P plants. Conversely, the percentage of root biomass was significantly greater in + P plants than in -P plants. Among the -P plants, those treated with 10 mg L⁻¹ TiO₂NPs presented the highest fresh root weight, whereas those treated with 50 mg L⁻¹ TiO₂NPs presented the highest dry root weight, significantly exceeding all the other treatments. In contrast, the plants treated with 100 mg L⁻¹ TiO₂NPs, regardless of their fertilization status, presented the lowest fresh and dry root weights. Interestingly, TiO₂NPs under P deficiency had a pronounced effect on the fresh and dry weights of the roots. The root biomass percentage followed an inverse trend, with the lowest significant values recorded in plants treated with 10 mg L⁻¹ TiO₂NPs, whereas the highest values were observed in plants treated with 100 mg L⁻¹, followed by those treated with 50 mg L⁻¹ TiO₂NPs, irrespective of phosphorus fertilization. These findings suggest that the lower concentration application narrowed the percentage between fresh and dry weight and vice versa at higher concentrations **(**Fig. 4**).**

### TiO₂NPs promote hormone synthesis and chlorophyll production

Two CKs have been identified in leaves as naturally occurring in plant tissues. Zeatin (ZT) and isopentenyl adenine (2iP) were analyzed to assess physiological changes in CK levels under phosphorus (P) deficiency and titanium dioxide (TiO₂) application. The concentration of 2iP in the leaves of all the treatments was greater than that of ZT. P deficiency significantly reduced ZT and 2iP levels, regardless of spraying application.

**Fig. 5:**
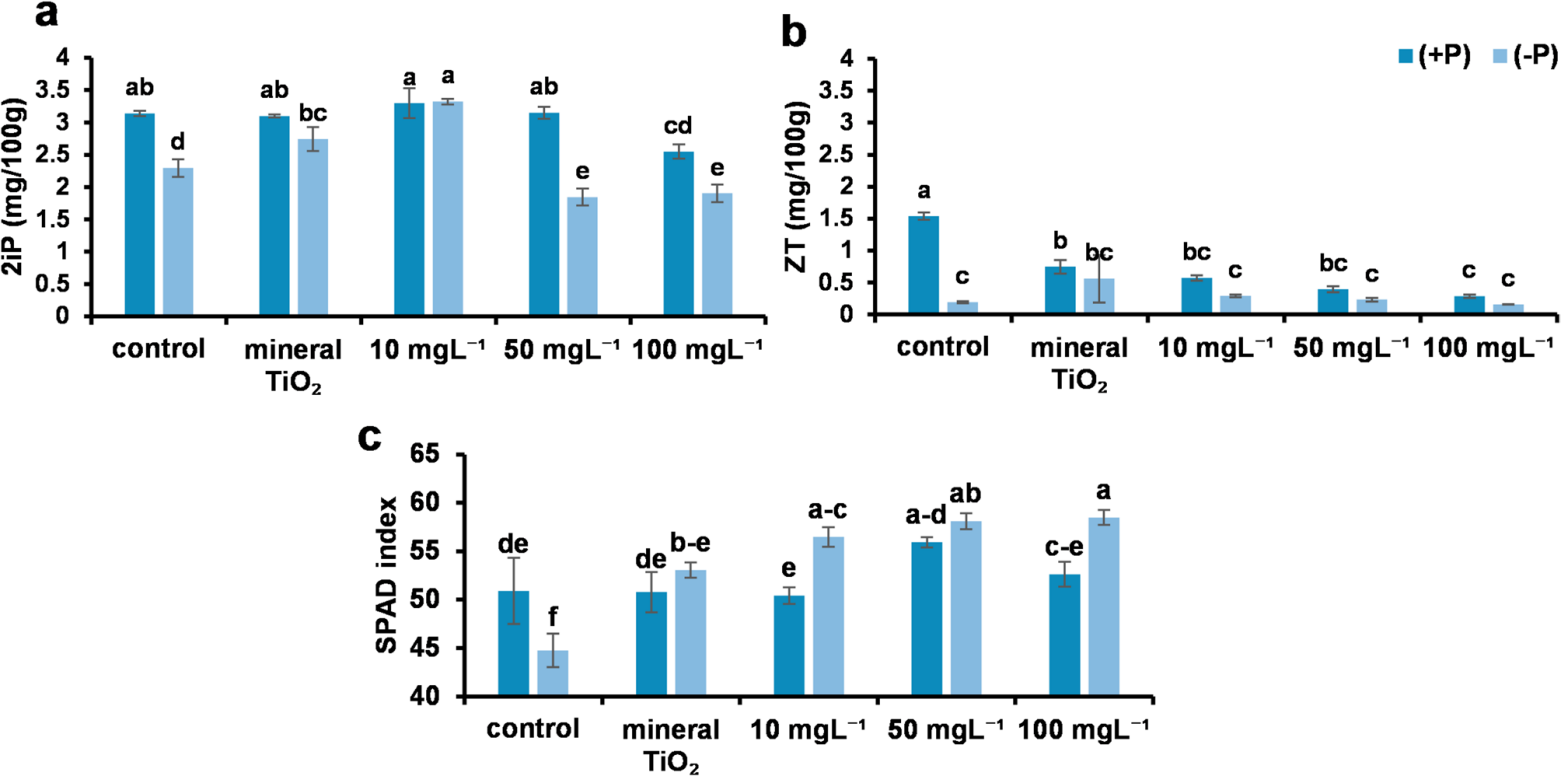
Represents the cytokinin CK) and chlorophyll contents in the leaves. Two natural forms of cytokinin hormones are (**a**) isopentyl adenine (2iP) per mg/100 g and (**b**) zeatin (ZT) per mg/100 g. (**c**) Leaf chlorophyll content (SPAD index). Different letters indicate significant differences between treatments at the significance level (*P* < 0.05). Error bars represent SEs.

**Fig. 6:**
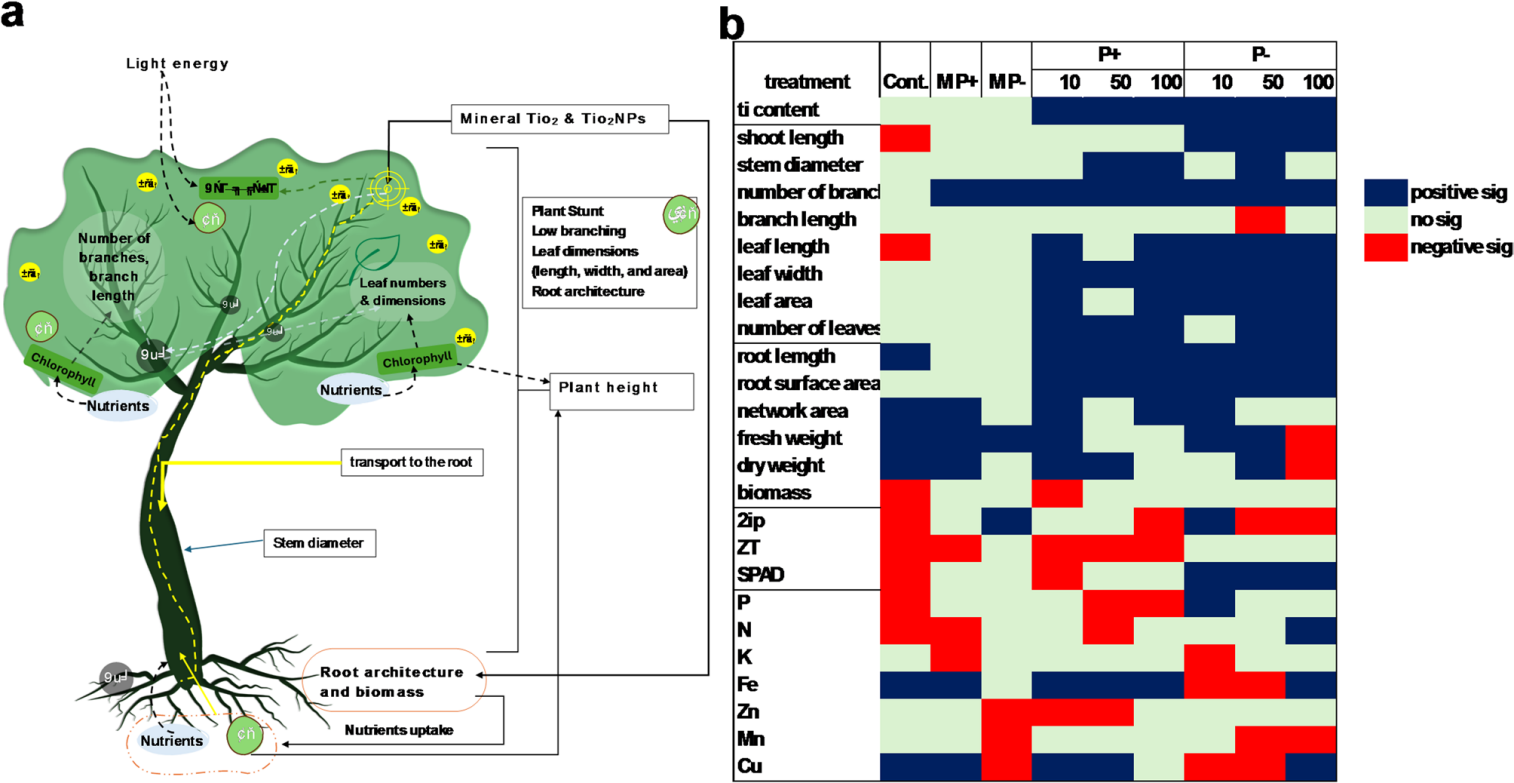
(**a**) an Illustration showing the mode of action of TiO₂NPs in regulating some physiological processes to alleviate P deficiency. The TiO₂NPs affect the CK content, which affects branching and other vegetative characteristics. TiO₂NPs improve root growth and development by changing root architecture, dry and fresh weights, and biomass and increasing the uptake of P and other nutrients. These specific nutrients affect chlorophyll, which affects plant height, stem diameter, leaf dimensions and counts. (**b**) Heatmap demonstrating the significant negative and positive effects of P and TiO₂NPs on all the measured parameters. MP- or MP + represents the mineral TiO₂ under P±, whereas 10, 50, and 100 represent these concentrations per mgL⁻¹. Cont. indicates the comparison of -P with + P in the control treatments.

The highest 2iP content was detected in the leaves of plants treated with 10 mg L⁻¹ TiO₂NPs under both ± P conditions, with no significant difference between + P and -P plants. Additionally, no significant difference was detected between + P plants treated with 10 mg L⁻¹ TiO₂NPs and + P control plants. However, a significant difference was detected between -P plants treated with 10 mg L⁻¹ TiO₂NPs and -P control plants, indicating that 10 mg L⁻¹ TiO₂NPs alleviated the reduction in 2iP levels. Notably, ±P plants sprayed with the highest TiO₂NP concentration presented the lowest 2iP content, which was significantly lower than that of ± P control plants. (Figs. 5 and 6).

The control plants presented the highest ZT levels, which were significantly different from those of all the other treatments. Among the -P and TiO₂NP treatments, the plants treated with 10 mg L⁻¹ TiO₂NPs presented the highest ZT content, but the ZT content did not significantly differ from that of the other -P treatments or the control. A significant difference in ZT content was observed between the -P and + P control plants, suggesting that the latter (Figs. 5 and 6).

With respect to the chlorophyll content in leaves, P deficiency significantly reduced chlorophyll levels. TiO₂NPs significantly alleviated this reduction; however, no significant differences were observed among 10, 50, and 100 mg L⁻¹ TiO₂NPs under -P conditions. Similarly, no significant differences were found between TiO₂NP-treated plants and control plants under + P conditions.

The effect of TiO₂ NPs on chlorophyll SPAD values was evaluated under both phosphorus availability (+ P) and phosphorus deficiency (-P). Under + P conditions, 10 mg L⁻¹ TiO₂ NPs increased chlorophyll SPAD values by 0.3% compared to the control (*P* > 0.05), while 50 mg L⁻¹ TiO₂ NPs significantly increased chlorophyll SPAD values by 9.1% compared to the control (*P* < 0.05). The highest concentration of 100 mg L⁻¹ TiO₂ NPs resulted in a significant increase of 9.1% compared to the control (*P* < 0.05).

Under -P conditions, 10 mg L⁻¹ TiO₂ NPs significantly increased chlorophyll SPAD values by 25.9% compared to the control (*P* < 0.05). At 50 mg L⁻¹ TiO₂ NPs, the increase was 30.8%, and 100 mg L⁻¹ TiO₂ NPs showed the highest improvement with a 30.8% increase in chlorophyll SPAD values compared to the control (*P* < 0.05). (Figs. 5 and 6).

### TiO₂NPs promote phosphorus uptake in apples under phosphorus deficiency conditions

The application of TiO₂ nanoparticles (NPs) had a notable effect on nutrient uptake, particularly under phosphorus-deficient (-P) conditions. The phosphorus content in the leaves was significantly greater in the + P treatment than in the -P treatment in the control group. Regardless of phosphorus fertilization, plants treated with 10 mg L⁻¹ TiO₂ NPs presented the highest leaf phosphorus (P) content, whereas the lowest P content was observed in plants treated with 50 and 100 mg L⁻¹ TiO₂ NPs. These findings suggest that high concentrations of TiO₂ NPs may have a negative or toxic effect on P absorption and allocation.

Under + P fertilization, there was no significant difference in the leaf P content between the control treatment and the 10 mg L⁻¹ TiO₂ NP treatment. In contrast, higher concentrations of TiO₂ NPs (50 and 100 mg L⁻¹) significantly reduced the leaf P content. Under -P conditions, the application of 10 mg L⁻¹ TiO₂ NPs significantly alleviated the reduction in P levels observed between the -P and + P control treatments. However, no significant differences were found between the control and the treatments with 50 and 100 mg L⁻¹ TiO₂ NPs or even the mineral TiO₂. These findings suggest that a lower concentration (10 mg L⁻¹) of TiO₂ NPs has a promising effect on alleviating P deficiency, whereas higher concentrations (50 and 100 mg L⁻¹) have adverse effects.

Nitrogen (N) uptake was generally lower in the control (-P) than in the control (+ P), indicating that phosphorus deficiency negatively affected N absorption, possibly due to its impact on chlorophyll and CK contents. However, the TiO₂NP treatments significantly improved the N levels in the -P treatment compared with those in the control, with the highest recorded values. Notably, there were no significant differences among 10, 50 and 100 mg L⁻¹. Under + P conditions, TiO₂NPs had variable effects but did not significantly outperform the control.

For potassium (K), an interesting trend was observed: the K content of the control (-P) did not significantly differ from that of the control (+ P), suggesting that phosphorus deficiency did not affect K absorption. Higher concentrations of TiO₂NPs (50 and 100 mg L⁻¹) maintained or slightly improved K levels but not significantly under -P. However, under + P, K levels generally remained lower across treatments, indicating that TiO₂NPs had a more pronounced effect on P-deficient conditions.

Iron (Fe) uptake was significantly enhanced by TiO₂NPs, especially 100 mg L⁻¹, under -P conditions, where the highest Fe content was recorded. Under + P conditions, Fe levels were lower, but TiO₂NPs still promoted a significant increase over the control. These findings suggest that TiO₂NPs may play a role in increasing Fe availability in plants. Similarly, zinc (Zn) uptake was greater under -P than + P; moreover, there were no significant differences between TiO₂NPs, particularly under -P conditions.

In contrast, manganese (Mn) uptake exhibited a different trend. Under -P conditions, TiO₂NP treatments generally reduced Mn levels, whereas under + P, Mn uptake remained stable across treatments. This suggests that TiO₂ NPs may influence Mn transport mechanisms differently depending on phosphorus availability.

Finally, TiO₂NPs under + P conditions significantly increased copper (Cu) uptake. Interestingly, under -P conditions, 100 mg L⁻¹ TiO₂NPs significantly increased Cu in leaves, whereas 50 mg L⁻¹ TiO₂NPs significantly increased Cu uptake **(**Fig. 6, Table 1**)**.

**Table 1:** Effect of titanium dioxide nano particles on leaf mineral content of MM106 Apple plants after tio₂ application under ± P conditions.

	leaf mineral content					
	Control	Normal	10 mgL⁻¹	50 mgL⁻¹	100 mgL⁻¹	Mean
	Phosphorus (P)					
(+ P)	1.00b ± 0.00	0.95b ± 0.00	0.87bc ± 5.55E-17	0.64d ± 0.00	0.73 cd ± 0.00	0.838B ± 0.027
(-P)	0.76 cd ± 0.00	0.70 cd ± 0.00	1.37a ± 0.00	0.87bc ± 5.55E-17	0.70 cd ± 0.00	0.880 A ± 0.052
Mean	0.88B ± 0.04	0.83 C ± 0.0417	1.12 A ± 0.0833	0.76 C ± 0.0383	0.72 C ± 0.005	-
	Nitrogen (N)					
(+ P)	3.63a ± 0.044	2.95c ± 0.139	3.28a-c ± 0.183	3.09bc ± 0.179	3.19a-c ± 0.088	3.23 A ± 0.073
(-P)	3.11bc ± 0.161	3.37a-c ± 0.214	3.54ab ± 0.161	3.50ab ± 0.120	3.59a ± 0.054	3.42 A ± 0.071
Mean	3.37 A ± 0.118	3.16 A ± 0.139	3.41 A ± 0.123	3.29 A ± 0.123	3.39 A ± 0.082	-
	Potassium (K)					
(+ P)	0.89 cd ± 0.010	0.80e ± 0.018	0.86de ± 0.014	0.85de ± 0.036	0.91b-d ± 0.016	0.86B ± 0.011
(-P)	0.95a-c ± 0.008	0.93bc ± 0.033	0.86de ± 0.002	0.99a ± 0.017	0.96ab ± 0.013	0.94 A ± 0.012
Mean	0.92 A ± 0.0116	0.86B ± 0.0278	0.86B ± 0.0067	0.92 A ± 0.0297	0.93 A ± 0.0132	-
	Iron (Fe)					
(+ P)	895.00 g ± 4.10	1283.20 cd ± 66.32	1094.40f ± 27.04	1118.00ef ± 30.27	1217.20de ± 19.18	1121.56B ± 30.70
(-P)	1398.80b ± 65.18	1347.20bc ± 10.11	1114.80ef ± 4.92	1267.60 cd ± 17.42	1668.80a ± 64.99	1359.44 A ± 40.96
Mean	1146.9CD ± 89.43	1315.20B ± 33.38	1104.60D ± 13.40	1192.80 C ± 29.88	1443.00 A ± 81.77	
	Zinc (Zn)					
(+ P)	308.00ab ± 9.80	294.60b-d ± 4.38	274.80 cd ± 19.65	269.60d ± 24.77	286.40b-d ± 16.28	286.68 A ± 7.34
(-P)	327.60a ± 5.04	296.40b-d ± 8.08	324.80a ± 0.80	301.20a-c ± 6.25	326.00a ± 0.89	315.20 A ± 3.46
Mean	317.80 A ± 6.14	295.50BC ± 4.34	299.80 A-C ± 12.47	285.40 C ± 13.14	306.20AB ± 10.13	
	Manganese (Mn)					
(+ P)	309.6a-c ± 8.33	350.00a ± 0.63	314.40a-c ± 3.43	325.60ab ± 5.88	328.00 ab ± 2.45	325.52 A ± 3.50
(-P)	334.80ab ± 2.94	256.80d ± 27.43	298.00bc ± 26.94	250.00d ± 4.90	276.00 cd ± 7.35	283.12B ± 9.57
Mean	322.20 A ± 5.91	303.40AB ± 20.21	306.20AB ± 13.09	287.80B ± 13.11	302.00AB ± 9.40	**-**
	Copper (Cu)					
(+ P)	50.80 cd ± 0.49	53.20a ± 0.49	52.00b ± 0.00	52.00b ± 0.00	51.20bc ± 0.49	51.84 A ± 0.23
(-P)	52.00b ± 0.00	50.00d ± 0.00	50.00d ± 0.00	50.80 cd ± 0.49	53.20a ± 0.49	51.20 A ± 0.28
Mean	51.40B ± 0.31	51.60AB ± 0.58	51.00B ± 0.33	51.40B ± 0.31	52.20 A ± 0.47	-

The statistical analyses are based on each Treatment and the interaction between treatments. The values of the different lowercase letters are significantly different at the and 0.05 probability levels, ± SE the uppercase letters show the significance difference between means for the phosphorus factor and the application of control and TiO₂ (NPs or Normal. (+ P) is the normal phosphorus fertilization (-P) is the phosphorus deficiency. Mineral TiO₂ of the titanium dioxide in the normal sort at 100 mgL⁻¹, while TiO₂NPs is the nano particles of titanium dioxide at 10,50, and 100 mgL⁻¹

## Discussion

The application of TiO₂NPs significantly alleviated phosphorus deficiency symptoms in apple plants, as evidenced by improved growth, higher chlorophyll content, and enhanced P accumulation. These findings are consistent with earlier reports suggesting that TiO₂NPs can play a beneficial role in improving nutrient acquisition and stress tolerance in plants. P deficiency generally has many impacts on plants^51^ and fruit trees, especially on apple trees. Plants with P deficiency initiate many physiological reactions, which are reflected in their biochemical contents in tissues and, subsequently, vegetative and root characteristics, in addition to improvements in the absorption of P and other nutrients^4,10,22,51,52^. In this study, P deficiency negatively affected vegetative and root feature shoot length, leaf length, leaf dimension and area, root fresh and dry weight, root biomass%, CK content, and chlorophyll content. P deficiency decreased not only the P leaf content but also the N leaf content, possibly because of changes in root characteristics and biochemical conditions^5,58,59^ which consequently could affect absorption as well. These findings revealed that the absorption mechanisms themselves can be impacted by P deficiency. Factors such as metabolite and phytohormone contents, such as CK and chlorophyll contents, may affect P and N absorption. (as mentioned in Fig. 6) .

Mechanistically, TiO₂NPs appear to exert their positive effects through several pathways. First, the enhanced root growth observed under TiO₂NPs treatment likely contributed to increased soil exploration and phosphorus uptake. Titanium dioxide is known to promote root elongation and branching, which may lead to a greater root surface area and more efficient nutrient absorption.

Second, TiO₂NPs may influence phosphorus solubilization in the rhizosphere by modifying root exudation patterns or interacting with microbial communities involved in P mineralization^38,53^. Increased phosphatase activity under TiO₂NP treatment also suggests a role in enhancing the mobilization of inorganic phosphate from organic sources, thus increasing P bioavailability.

Furthermore, TiO₂NPs are known to improve photosynthetic efficiency and chlorophyll biosynthesis, which can support better energy metabolism under P-deficient conditions. Enhanced antioxidant enzyme activity (such as SOD, CAT, and POD) observed in the present study indicates that TiO₂NPs help mitigate oxidative stress induced by phosphorus deficiency, thus preserving cellular integrity and function.

Although the present study focused primarily on physiological and biochemical responses, it is worth noting that TiO₂NPs may also influence phosphorus uptake at the molecular level. Previous studies have indicated that phosphorus deficiency triggers the upregulation of specific phosphate transporter genes, such as *PHT1;1*,* PHT1;4*, and other members of the *PHT1* family. It is plausible that TiO₂NPs may indirectly modulate the expression of these genes by altering root architecture, oxidative balance, or rhizosphere conditions, thereby enhancing P acquisition. Further molecular studies are needed to validate these potential gene-level interactions and fully elucidate the underlying mechanisms^54^. The integration of physiological, biochemical, and molecular analyses will be essential to fully elucidate the mechanisms by which TiO₂NPs enhance P acquisition and tolerance. TiO₂ may enhance this response, either by triggering signaling cascades (e.g., involving reactive oxygen species or phytohormones) or by mimicking mild stress conditions that activate the phosphate starvation response (PSR) pathway.

These mechanisms, together with possible changes in root morphology and microbial interactions^31–35,55−57^, suggest that TiO₂ plays a multifaceted role in improving phosphorus acquisition and utilization under deficient conditions.

In general, foliar TiO₂NP application caused some changes in cell biochemical and stimulate CK and other metabolites. This changes in cooperation with CK subsequently affected the roots by changing their architecture and improving the uptake of P, and other nutrients^36,57^. This improvement in specific nutrients affects chlorophyll, which affects plant height, stem diameter, leaf dimensions, and counts. This might occur through the transport of TiO₂NPs to the root system (underground parts), changing the biochemical compounds and subsequently altering root development. On the other hand, TiO₂NPs significantly affect vegetative features. These morphological and physiological impacts improve the ability of roots to absorb nutrients under normal conditions and under phosphorus (P) deficiency. (as mentioned in Fig. 6).

TiO₂NPs not only affect nutrient absorption and allocation but also affect biochemical metabolites and hormones such as cytokines. TiO₂NPs, have impacts in changing the soil pH and organic molecules, which were reported in other studies^57^. This would stimulate the composition of biochemicals in the roots and rhizosphere and consequently change the fresh weight and dry weight of the roots. Biochemicals may then be secreted from the roots or rhizosphere to the soil to improve root absorption^11,12^. These factors comprehensively affect plant growth and development, particularly under P deficiency.

Typically, P deficiency has a profound impact on CK levels and signaling in plants. Under P deficiency, CK biosynthesis is generally reduced^58,60−62^ particularly in the roots, leading to lower CK transport to the shoots, which was in the same trend as the study results. This hormonal imbalance negatively affects shoot development while promoting root growth, an adaptive strategy to enhance P uptake^59^. Additionally, reduced CK levels downregulate CK signaling genes^58,60−63^ leading to significant modifications in root architecture^58^such as increased root hair and lateral root formation^56,58,60,61,64^ length of different root diameters, root hair density, Root surface and network area.

However, when the plant were applied with low levels of TiO₂NPs (10 and 50 mg L⁻¹), CK levels typically recover, restoring an adjustment in root-to-shoot development and supporting both root and shoot growth. Understanding these hormonal adjustments provides insight into how plants respond to nutrient stress and may help in developing strategies to mitigate P deficiency effects^59^. This dynamic regulation highlights the critical role of CK in mediating root architecture responses to nutrient availability^59^.

The observed CK moderate concentration suggests a potential balance between inhibitory and stimulatory effects on root and shoot growth under phosphorus (P) deficiency^65^. Previous studies indicated that while high CK levels generally suppress root development^66,67^ moderate concentrations may promote root growth and development. In this context, the measured CK level could contribute to influence root development, enhancing the plant’s ability to explore soil for phosphorus.

Additionally, CK interacts with auxin and ethylene^56,57,65,68,69^ two key regulators of root and shoot development. Under normal P- deficiency conditions, reduced CK levels allow auxin to accumulate in plant cells^58,69^ stimulating changing the root and shoot growth and development^69^. However, if CK levels drop too low, it may impair auxin transport or signaling, leading to suppressing growth^69,70^. Conversely, elevated CK levels in our study may have facilitated auxin redistribution, indirectly promoting growth and development. Further investigation into CK’s interaction with auxin, ethylene, and P- deficiency signaling pathways will provide deeper insights into the hormonal regulation of root hair development in nutrient-limited conditions.

The lowest concentration of TiO₂NPs (10 mgL⁻¹) improved root characteristics such as length and surface area across the different root diameter ranges. The same occurred for the fresh and dry weights of the roots, whereas the lower and medium concentrations resulted in increases in the fresh and dry weights of the roots. The changes in the root characteristics may be due to the effect of TiO₂NPs on the rhizosphere^33^. The lower concentration of TiO₂NPs increased the length of the roots and the surface area for all the diameters recorded under P deficiency, whereas there was a significant difference at the medium and high concentrations. These findings reveal that a relatively low concentration of TiO₂NPs affects both the root and rhizosphere reactions to improve the absorption of P under P deficiency (as mentioned in Fig. 6) .

The physiological changes caused by the application of TiO₂NPs alleviated the decrease in leaf chlorophyll content under P deficiency^10^. The combination of plant length, leaf area, and chlorophyll increase caused by TiO₂NPs may consequently play a role in increasing the carbohydrate content for transport to the roots and improving their development characteristics(as mentioned in Fig. 6) .

The results reveal that TiO₂ NPs, particularly at higher concentrations, can significantly improve chlorophyll SPAD values under both phosphorus conditions. Under + P conditions, the impact of TiO₂ NPs was moderate, with 50 mg L⁻¹ and 100 mg L⁻¹ TiO₂ NPs both showing a statistically significant increase in chlorophyll SPAD values compared to the control. However, the increase was relatively small for 10 mg L⁻¹ TiO₂ NPs, suggesting that higher concentrations of TiO₂ NPs may be more effective under optimal phosphorus conditions.

Under -P conditions, TiO₂ NPs showed a much stronger effect, with 10 mg L⁻¹, 50 mg L⁻¹, and 100 mg L⁻¹ TiO₂ NPs all significantly increasing chlorophyll SPAD values compared to the control. The highest concentrations (50 mg L⁻¹ and 100 mg L⁻¹) showed the greatest improvement, indicating that TiO₂ NPs can alleviate phosphorus deficiency symptoms by enhancing chlorophyll synthesis, particularly under nutrient-limited conditions. These results suggest that TiO₂ NPs could potentially serve as a beneficial treatment for improving plant chlorophyll content under phosphorus stress.

Interestingly, this study confirms the findings of other studies in that there are toxic or negative effects of TiO₂NPs at relatively high concentrations on several characteristics^36,71^, such as the fresh and dry weights of roots, CK contents, nutrient absorption, and root network area. In contrast, this effect did not clearly appear for some characteristics, such as shoot length, stem diameter, number of branches, leaf dimensions (length, width, or area), number of leaves per plant, root length, and surface area^36^. It is recommended that the use of TiO₂NPs at lower concentrations is better than that at higher concentrations^36,71^. A relatively low concentration of TiO₂NPs increased the level of CK in the leaves; however, the medium and relatively high concentrations did not significantly affect the CK under P deficiency^36^. The negative effect of P deficiency on CK clearly appeared.

Notably, there was a slight fluctuation between the three different concentrations of TiO₂NPs in terms of increasing and decreasing morphological and physiological characteristics. Moreover, a relatively low concentration of TiO₂NPs (10 mg L⁻¹) clearly increased the P in leaves under P deficiency. These findings reflected the impact of TiO₂NPs on increasing P in roots. This finding was in parallel with many studies, which reported that the application of titanium increases P uptake or Phyto availability^31,33^.

There is still a gap in knowledge concerning the mechanism of TiO₂NPs to change CKs and other metabolites under different stresses; however, some studies have investigated the effects of P deficiency on CKs. Because plants are important fruit trees around the world^12^, more research on the role of TiO₂NPs in the physiological and morphological impacts of these materials is needed. The combination of P deficiency, TiO₂NPs, and CK in this study revealed the physiological effects of TiO₂NPs on CK under P deficiency. Zeatin and isopentyl adenine, as natural sources of CK, were recorded. The lowest concentration (10 mg L⁻¹) alleviated the decrease in CK in both Zeatin and Isopentyl Adenine sources under P deficiency.

In conclusion, TiO₂NPs alleviate phosphorus deficiency in apple through a multifaceted mechanism involving root morphological changes, enhanced phosphatase activity, improved photosynthetic efficiency, and activation of antioxidant defense systems. These findings provide valuable insights into the potential application of TiO₂NPs in sustainable apple production under low-P conditions.

## Conclusion

TiO₂NPs play a significant role in promoting apple plant growth and development under both normal and P-deficient conditions. TiO₂NPs alleviated P deficiency symptoms, which included plant stunts, low branching, a small leaf area, and root architecture characteristics, when TiO₂NPs stimulated CK formation to increase branching parameters and stimulating physiological mechanisms to improve plant growth. It participated in modifying vegetative and root growth and development TiO₂NPs increased CK, chlorophyll, and other vegetative parameters. TiO₂NPs are transported to roots to change the root architecture and biomass content to promote the uptake of P and other nutrients to help plants overcome P deficiency. Further studies are needed to determine the precise mechanisms of TiO₂NP action and establish safe, effective application protocols for sustainable agricultural use.

This study provides new insights into how TiO₂ nanoparticles can interact with cytokinin pathways to alleviate phosphorus deficiency in apple trees. The findings contribute to a relatively unexplored area, offering a promising avenue for improving nutrient efficiency in fruit crop management through nanotechnology and hormonal modulation.

## Electronic supplementary material

Below is the link to the electronic supplementary material.

Supplementary Material 1

## Data Availability

The datasets used, generated and/or analyzed during the current study are available from the corresponding author on reasonable request.
