# Supplementary material for: Titanium dioxide nanoparticles alleviate phosphorus deficiency stress in apple plants
Source: Sci Rep. 2025 Jul 8;15:24443. doi: 10.1038/s41598-025-07987-3 (PMC12238376; doi:10.1038/s41598-025-07987-3)
Supplement: Supplementary file 1 — Supplementary Material 1 [file 41598_2025_7987_MOESM1_ESM.pdf]

Root analysis

Supplementary Table 1: Effect of titanium dioxide nano particles total root surface area cm^2 of MM106 apple under phosphorus deficiency

treatment	control	Mineral TiO_2	TiO_2NPs			Means
		100 mgL^{-1}	10 mgL^{-1}	50 mgL^{-1}	100 mgL^{-1}	
(+P)	3250.38d	4346.08b	4776.96ab	3258.11d	4108.52bc	3948.01Bˋ
(-P)	4343.09bc	4016.48c	5403.10a	4199.49bc	3838.30cd	4360.09Aˋ
Means	3796.73B	4181.28B	5090.03A	3728.80B	3973.41B	

Note: The statistical analyses are based on each Treatment and the interaction between treatments. The values of the different lowercase letters are significantly different at the and 0.05 probability levels, the uppercase letters show the significance difference between means for the phosphorus factor and the application of control and TiO2 (NPs or Normal. (+P) is the normal phosphorus fertilization (-P) is the phosphorus deficiency. Mineral Tio2 of the titanium dioxide in the normal sort at 100 mgL^{-1}, while TiO2NPs is the nano particles of titanium dioxide at 10,50, and 100 mgL^{-1}

Supplementary Table 2: Effect of titanium dioxide nano particles on root length per cm for range 2mm diameter of MM106 apple under phosphorus deficiency

treatment	control	Mineral TiO_2	TiO_2NPs			Means
		100 mgL^{-1}	10 mgL^{-1}	50 mgL^{-1}	100 mgL^{-1}	
(+P)	451.15b-d	597.45b	483.70b-d	419.93b-d	466.32b-d	483.71Aˋ
(-P)	411.33b-d	309.65cd	948.08a	526.28bc	274.70d	494.01Aˋ
Means	431.24B	453.55B	715.89A	473.10B	370.51B	

Note: The statistical analyses are based on each Treatment and the interaction between treatments. The values of the different lowercase letters are significantly different at the and 0.05 probability levels, the uppercase letters show the significance difference between means for the phosphorus factor and the application of control and TiO2 (NPs or Normal. (+P) is the normal phosphorus fertilization (-P) is the phosphorus deficiency. Mineral Tio2 of the titanium dioxide in the normal sort at 100 mgL^{-1}, while TiO2NPs is the nano particles of titanium dioxide at 10,50, and 100 mgL^{-1}

Supplementary Table 3: Effect of titanium dioxide nano particles on root length per cm for range 3mm diameter of MM106 apple under phosphorus deficiency

treatment	control	Mineral TiO_2	TiO_2NPs			Means
		100 mgL^{-1}	10 mgL^{-1}	50 mgL^{-1}	100 mgL^{-1}	
(+P)	159.24bc	201.58b	162.38bc	166.53bc	193.00b	176.55Aˋ
(-P)	152.37bc	134.90bc	278.12a	206.84b	98.45c	174.13Aˋ
Means	155.80B	168.24AB	220.25A	186.68AB	145.73B	

Note: The statistical analyses are based on each Treatment and the interaction between treatments. The values of the different lowercase letters are significantly different at the and 0.05 probability levels, the uppercase letters show the significance difference between means for the phosphorus factor and the application of control and TiO2 (NPs or Normal. (+P) is the normal phosphorus fertilization (-P) is the phosphorus deficiency. Mineral Tio2 of the titanium dioxide in the normal sort at 100 mgL^{-1}, while TiO2NPs is the nano particles of titanium dioxide at 10,50, and 100 mgL^{-1}

Supplementary Table 4: Effect of titanium dioxide nano particles on root length per cm for range 4mm diameter of MM106 apple under phosphorus deficiency

treatment	control	Mineral TiO_2	TiO_2NPs			Means
		100 mgL^{-1}	10 mgL^{-1}	50 mgL^{-1}	100 mgL^{-1}	
(+P)	227.59b-d	341.14bc	286.32bc	297.62bc	272.46bc	285.03Aˋ
(-P)	247.18b-d	213.66cd	521.97a	347.87b	139.10d	293.96Aˋ
Means	237.38C	277.40BC	404.15A	322.74B	205.78C	

Note: The statistical analyses are based on each Treatment and the interaction between treatments. The values of the different lowercase letters are significantly different at the and 0.05 probability levels, the uppercase letters show the significance difference between means for the phosphorus factor and the application of control and TiO2 (NPs or Normal. (+P) is the normal phosphorus fertilization (-P) is the phosphorus deficiency. Mineral Tio2 of the titanium dioxide in the normal sort at 100 mgL^{-1}, while TiO2NPs is the nano particles of titanium dioxide at 10,50, and 100 mgL^{-1}

Supplementary Table 5: Effect of titanium dioxide nano particles on root length per cm for range 5mm diameter of MM106 apple under phosphorus deficiency

treatment	control	Mineral TiO_2	TiO_2NPs			Means
		100 mgL^{-1}	10 mgL^{-1}	50 mgL^{-1}	100 mgL^{-1}	
(+P)	123.41bc	163.22b	135.74bc	159.18b	168.73b	150.06Aˋ
(-P)	144.80bc	126.03bc	229.23a	185.23ab	91.26c	155.31Aˋ
Means	134.11BC	144.63A-C	182.49A	172.20AB	129.99C	

Note: The statistical analyses are based on each Treatment and the interaction between treatments. The values of the different lowercase letters are significantly different at the and 0.05 probability levels, the uppercase letters show the significance difference between means for the phosphorus factor and the application of control and TiO2 (NPs or Normal. (+P) is the normal phosphorus fertilization (-P) is the phosphorus deficiency. Mineral Tio2 of the titanium dioxide in the normal sort at 100 mgL^{-1}, while TiO2NPs is the nano particles of titanium dioxide at 10,50, and 100 mgL^{-1}

Supplementary Table 6: Effect of titanium dioxide nano particles on root length per cm for range 6mm diameter of MM106 apple under phosphorus deficiency

treatment	control	Mineral TiO_2	TiO_2NPs			Means
		100 mgL^{-1}	10 mgL^{-1}	50 mgL^{-1}	100 mgL^{-1}	
(+P)	1995.00d	2457.33b-d	2641.52b	2126.51cd	2510.67bc	2346.20Bˋ
(-P)	2431.30b-d	2267.13b-d	3347.81a	2546.33bc	2132.73cd	2545.06Aˋ
Means	2213.15B	2362.23B	2994.66A	2336.42B	2321.70B	

Note: The statistical analyses are based on each Treatment and the interaction between treatments. The values of the different lowercase letters are significantly different at the and 0.05 probability levels, the uppercase letters show the significance difference between means for the phosphorus factor and the application of control and TiO2 (NPs or Normal. (+P) is the normal phosphorus fertilization (-P) is the phosphorus deficiency. Mineral Tio2 of the titanium dioxide in the normal sort at 100 mgL^{-1}, while TiO2NPs is the nano particles of titanium dioxide at 10,50, and 100 mgL^{-1}

Supplementary Table 7: Effect of titanium dioxide nano particles on surface Area per cm^2 range 2mm diameter of MM106 apple under phosphorus deficiency

treatment	control	Mineral TiO_2	TiO_2NPs			Means
		100 mgL^{-1}	10 mgL^{-1}	50 mgL^{-1}	100 mgL^{-1}	
(+P)	283.47b-d	375.39b	303.92b-d	263.85b-d	293.00b-d	303.92Aˋ
(-P)	258.45b-d	194.56cd	595.70a	330.67bc	172.60d	310.40Aˋ
Means	270.96B	284.97B	449.81A	297.26B	232.80B	

Note: The statistical analyses are based on each Treatment and the interaction between treatments. The values of the different lowercase letters are significantly different at the and 0.05 probability levels, the uppercase letters show the significance difference between means for the phosphorus factor and the application of control and TiO2 (NPs or Normal. (+P) is the normal phosphorus fertilization (-P) is the phosphorus deficiency. Mineral Tio2 of the titanium dioxide in the normal sort at 100 mgL^{-1}, while TiO2NPs is the nano particles of titanium dioxide at 10,50, and 100 mgL^{-1}

Supplementary Table 8: Effect of titanium dioxide nano particles on surface Area per cm2 range 3mm diameter of MM106 apple under phosphorus deficiency

treatment	control	Mineral TiO_2	TiO_2NPs			Means
		100 mgL^{-1}	10 mgL^{-1}	50 mgL^{-1}	100 mgL^{-1}	
(+P)	141.49bc	179.12b	144.29bc	147.97bc	171.50b	156.87Aˋ
(-P)	135.39bc	119.87bc	247.13a	183.79b	87.48c	154.73Aˋ
Means	138.44B	149.50AB	195.71A	165.88AB	129.49B	

Note: The statistical analyses are based on each Treatment and the interaction between treatments. The values of the different lowercase letters are significantly different at the and 0.05 probability levels, the uppercase letters show the significance difference between means for the phosphorus factor and the application of control and TiO2 (NPs or Normal. (+P) is the normal phosphorus fertilization (-P) is the phosphorus deficiency. Mineral Tio2 of the titanium dioxide in the normal sort at 100 mgL^{-1}, while TiO2NPs is the nano particles of titanium dioxide at 10,50, and 100 mgL^{-1}

Supplementary Table 9 Effect of titanium dioxide nano particles on surface Area per cm2 range 4mm diameter of MM106 apple under phosphorus deficiency

treatment	control	Mineral TiO_2	TiO_2NPs			Means
		100 mgL^{-1}	10 mgL^{-1}	50 mgL^{-1}	100 mgL^{-1}	
(+P)	286.00b-d	428.69bc	359.81bc	373.99bc	342.39bc	358.17Aˋ
(-P)	310.62b-d	268.50cd	655.93a	437.15b	174.80d	369.40Aˋ
Means	298.31C	348.59BC	507.87A	405.57B	258.59C	

Note: The statistical analyses are based on each Treatment and the interaction between treatments. The values of the different lowercase letters are significantly different at the and 0.05 probability levels, the uppercase letters show the significance difference between means for the phosphorus factor and the application of control and TiO2 (NPs or Normal. (+P) is the normal phosphorus fertilization (-P) is the phosphorus deficiency. Mineral Tio2 of the titanium dioxide in the normal sort at 100 mgL^{-1}, while TiO2NPs is the nano particles of titanium dioxide at 10,50, and 100 mgL^{-1}

Supplementary Table 10: Effect of titanium dioxide nano particles on surface Area per cm2 range 5mm diameter of MM106 apple under phosphorus deficiency after treatment of TiO_2

treatment	control	Mineral TiO_2	TiO_2NPs			Means
		100 mgL^{-1}	10 mgL^{-1}	50 mgL^{-1}	100 mgL^{-1}	

(+P)	173.39bc	229.32b	190.71bc	223.64b	237.06b	210.83Aˋ
(-P)	203.44bc	177.07bc	322.06a	260.24ab	128.21c	218.20Aˋ
Means	188.41BC	203.20A-C	256.39A	241.94AB	182.63C	

Note: The statistical analyses are based on each Treatment and the interaction between treatments. The values of the different lowercase letters are significantly different at the and 0.05 probability levels, the uppercase letters show the significance difference between means for the phosphorus factor and the application of control and TiO2 (NPs or Normal. (+P) is the normal phosphorus fertilization (-P) is the phosphorus deficiency. Mineral Tio2 of the titanium dioxide in the normal sort at 100 mgL^{-1}, while TiO2NPs is the nano particles of titanium dioxide at 10,50, and 100 mgL^{-1}

Supplementary Table 11: Effect of titanium dioxide nano particles on surface Area per cm2 range 6mm diameter of MM106 apple under phosphorus deficiency

treatment	control	Mineral TiO_2	TiO_2NPs			Means
		100 mgL^{-1}	10 mgL^{-1}	50 mgL^{-1}	100 mgL^{-1}	
(+P)	40739.00c	46443.81bc	61716.76a	38629.51c	54784.12ab	48462.64Bˋ
(-P)	57336.68ab	59756.77a	56446.08ab	53751.85ab	64491.14a	58356.50Aˋ
Means	49037.84B	53100.29AB	59081.42A	46190.68B	59637.63A	

Note: The statistical analyses are based on each Treatment and the interaction between treatments. The values of the different lowercase letters are significantly different at the and 0.05 probability levels, the uppercase letters show the significance difference between means for the phosphorus factor and the application of control and TiO2 (NPs or Normal. (+P) is the normal phosphorus fertilization (-P) is the phosphorus deficiency. Mineral Tio2 of the titanium dioxide in the normal sort at 100 mgL^{-1}, while TiO2NPs is the nano particles of titanium dioxide at 10,50, and 100 mgL^{-1}
